# Socio-demographic characteristics and tobacco use among the adults in urban slums of Dhaka, Bangladesh

**DOI:** 10.1186/s12971-017-0131-1

**Published:** 2017-05-05

**Authors:** Nusrat Nausheen Khandker, Tuhin Biswas, Abdullah Nurus Salam Khan, Enamul Hasib, Lal B Rawal

**Affiliations:** 1 James P Grant School of Public Health, BRAC University, Dhaka, Bangladesh; 2 Health Systems and Population Studies Division, icddr, b Level 5, 68 Shaheed Tajuddin Ahmed Sharani, Mohakhali, Dhaka, 1212 Bangladesh

**Keywords:** Tobacco use, Urban slum, Dhaka, Bangladesh

## Abstract

**Background:**

Use of tobacco has become one of the major causes of premature deaths in most developing countries, including Bangladesh. The poorest and most disadvantaged populations, such as those living in slums, are considered to be extremely vulnerable to non-communicable diseases and their risk factors, especially tobacco use. The objective of this study was to assess the current status of tobacco consumption among slum dwellers and its association with socio-demographic factors.

**Methods:**

A cross-sectional study was conducted in three slums of Dhaka city. Information about tobacco use as well as socio-demographic characteristics was collected from adult slum dwellers via face to face interviews using WHO STEPS questionnaire.

**Result:**

Overall proportion of smoking, smokeless tobacco consumption and dual use of tobacco was 35% [95% CI: 31.6-39.8], 40.6% [95% CI: 36.5–45.2] and 12% [95% CI: 9.3–15.0] respectively. Elderly people (55–64 years) were more likely to smoke (OR: 2.34, 95% CI: 1.21–4.49) than younger people (aged 25–34 years). On the other hand, those who had no schooling history (OR: 2.95, 95% CI: 1.66–5.25) were more likely to consume smokeless tobacco than those who had higher education (secondary or above). At the same time, manual workers were more likely to indulge in dual use of tobacco (OR: 5.17, 95% CI: 2.82–9.48) as compared to non-manual workers.

**Conclusion:**

The urban slum population of Dhaka city has a high prevalence of tobacco use, which increases their likelihood of developing non-communicable diseases. Proper attention needs to be directed towards addressing the risk factors related to non-communicable diseases within this vulnerable population.

## Background

Tobacco use has been unanimously regarded as one of the leading preventable causes of death and disability worldwide [1]. About 6 million people die each year as a result of tobacco use [2]. According to the Global Burden of Disease report (2010), consumption of tobacco products leads to premature deaths as well as disability accounting for 6.9% of years of life lost and 5.5% of disability-adjusted life-years (DALYs) respectively [3]. Tobacco use has also been recognized as one of the four behavioural risk factors responsible for causing non-communicable diseases (NCDs), especially the four major NCDs namely cardiovascular diseases, chronic respiratory diseases, type 2 diabetes mellitus and cancer [1]. The harms of tobacco use are not only limited to the user. Second-hand smoking (SHS) also has serious health consequences, and causes about 600,000 deaths worldwide [2].

It has been estimated that more than two-thirds of deaths due to tobacco consumption occur in the low and middle income countries [4]. The nations in South-East Asian Region (SEAR) have one of the highest burdens of tobacco-related morbidity and mortality. More than 1 million out of the 6 million tobacco-attributable deaths worldwide, occur in the SEAR countries [5]. Two vital issues that draw attention to the tobacco use pattern in the SEAR countries (especially India and Bangladesh) are the increased consumption of smokeless tobacco (SLT) and the use of bidis (hand-rolled cigarettes), which pose more of a threat to people’s health because of the high content of nicotine and other harmful chemicals [5].

Like other developing countries, Bangladesh too faces a huge burden of tobacco consumption and its related illnesses. The Global Adult Tobacco Survey (GATS), 2009, estimates that 43.3% of Bangladeshis (aged 15 and above) use tobacco in some form or the other [6]. Data from the NCD risk factor survey in Bangladesh showed an overall tobacco use prevalence of 54% among adults over 25 years of age [7]. Studies have shown this high prevalence to be linked with a higher morbidity and mortality due to tobacco use. A proportional mortality study revealed that in 2010, smoking-attributable mortality in Bangladeshi men aged between 25 to 69 years was about 25% [8]. Another study, published in 2013, echoes these findings and attributes 25% of the deaths in men and 7.6% of the deaths in females to smoking [9]. The same study also reports an increased risk of mortality from cancer and ischemic heart disease as well as an increase in all-cause mortality among men who smoked, as compared to their non-smoker counterparts [9].

Studies have suggested that the prevalence of tobacco use is high in poor people [10] and it has been found that people from households in the poorest quintiles in low and middle income countries such as Bangladesh, are twice more likely to smoke than the wealthier households [11]. In Bangladesh, the poorest people live in slums and account for about one-third of the urban population [12]. The urban slum dwellers are not only economically disadvantaged, but live in overcrowded settlements with low levels of sanitation and hygiene, open garbage disposal, lack of proper healthcare facilities and so on, which readily contribute to ill health and disease [12, 13]. These factors, along with changing behavioral norms related to NCD risk factors in developing countries [14], make the slum dwellers a high-risk population for tobacco use and development of NCDs. The International Tobacco Control (ITC) Bangladesh Survey revealed that the prevalence of tobacco use among slum-dwellers was much higher (78.8%) as compared to the rest of the urban population [15]. Studies conducted in neighboring countries like India also proclaimed higher rates of tobacco usage within their slum population [16, 17]

It can thus be estimated from national surveys and other studies conducted in similar contexts, that the slum population has a higher prevalence of tobacco use. However, there is a dearth of studies with special focus on disadvantaged populations such as the urban slum dwellers. Also, after the ITC Bangladesh Survey in 2010, there has been no routine surveillance on tobacco use. Rapid urbanization resulting in an increased slum population over the past few years, coupled with changing norms due to globalization have had an impact on the lifestyles of the people living in these areas. This makes it even more crucial to obtain up-to-date information on this vulnerable population, which can aid in assessing the magnitude of NCD risk and related problems among the slum dwellers. This study aimed to bring out the current picture of tobacco use among the adults residing in the urban slums of Dhaka and to examine any association between tobacco use and the socio-demographic characteristics of the respondents.

## Methods

### Study setting and population

A cross-sectional study was carried out in three purposively selected urban slums of Dhaka, namely Korail slum in Gulshan, Bhashantek slum in Mirpur and Rayerbazar slum in Dhanmondi. The study population comprised of adult slum dwellers (aged 25 to 64 years) residing in those slums. This age group has been recommended by WHO as the target age group for the survey of NCD risk factors [18]. Persons living in the selected slums for at least 6 months were included in the study. This study was a part of a larger study which also collected data on other NCD risk factors including alcohol consumption, food and vegetable intake, anthropometric and blood pressure measurement using the WHO STEPS Questionnaire and are in process of being published elsewhere. In order to align with the aim of the larger study and collect as comprehensive data as possible, the critically ill or bed-ridden persons, pregnant women and physically or mentally challenged individuals who were unable to participate in the study were excluded.

### Sampling strategy

Multi-stage cluster sampling was adopted, whereby households were selected as the sampling units. Using existing maps of the three slums, geographical clusters were identified based on road networks. The clusters were divided in such a manner that they were nearly equal on visual estimation. In order to obtain uniform samples from each slum, the total sample size (507, rounded off to 510 for the ease of calculations) was divided into three equal portions. From the clusters identified within one slum, two clusters were selected by simple random sampling (random number generation). Real time images of each cluster were obtained by using Google Earth and used as guides for sampling. Households were selected using systematic random sampling. Alternate households were approached and male and female respondents were also chosen from these households in an alternate manner. If there were more than one eligible member within a household, one respondent was selected randomly by lottery. Finally, 507 eligible participants completed the interview and physical measures were taken.

### Data collection process

Data was collected via face-to face interviews, using a structured questionnaire. The questionnaire was adopted from the Step 1 of WHO STEPS Questionnaire [19]. The demographic information section and individual sections on tobacco use were adopted with minor contextual modifications. A validated Bengali version of the WHO STEPS Questionnaire was consulted for translation of the tool into Bengali [7].

The participants were mainly asked about their consumption of “smoked tobacco” and “smokeless tobacco”. According to the NCD Global Monitoring Framework guidelines, “smoked tobacco products includes the consumption of cigarettes, bidis, cigars, cheroots, pipes, shisha (water pipes), fine‐cut smoking articles (roll‐your‐own), krekets, and any other form of smoked tobacco”[20]. On the other hand, “smokeless tobacco includes moist snuff, plug, creamy snuff, dissolvables, dry snuff, gul, loose leaf, red tooth powder, snus, chimo, gutkha, khaini, gudakhu, zarda, quiwam, dohra, tuibur, nasway, naas/naswar, shammah, betel quid, toombak, pan (betel quid), iq’mik, mishri, tapkeer, tombol and any other tobacco product that is sniffed, held in the mouth, or chewed” [20].

The data were collected by trained interviewers, which included physicians and trained field research assistants with a health science background. Data collectors were exclusively trained on anthropometric measurements and physical examination. The English version of the questionnaire was prepared, which was then translated into Bangla version once we had expert review and comments/feedback from the subject experts. The Bangla version questionnaire was back translated into English once the pilot testing was completed to establish the reliability and validity of the translation. The final pilot tested Bangla version questionnaire was used for data collection.

### Ethics approval

The ethics approval for conducting this study was obtained from the Ethical Review Committee of James P Grant School of Public Health, BRAC University, Bangladesh. During data collection, the research objectives and procedures were explained to the participants in Bengali. Due to the contextual setting where most of the participants were illiterate and because of  the sensitive nature of requesting thumbprints or signatures, verbal informed consent was taken from the participants before the interviews. The consent script was read out clearly by the interviewer at the beginning of each interview and their consent to participate or not to participate in the study was sought.

### Data management and quality control

Prior to the study, the questionnaire was pre-tested among slum adult populations residing in another slum area (Shattola slum in Mohakhali, Dhaka). Data collectors were trained extensively before going to the field. Demonstrations and mock-interviews were also carried out. Random checks of the interviews were performed to identify any non-response pattern or errors in data collection.

### Statistical analysis

Data was analyzed using the statistical software, IBM SPSS Statistics (version 20). 507 samples were used for overall analysis. As per the WHO STEPS Surveillance Data Analysis guideline, records having missing data for the age variable were dropped [19]. Non-responses for particular sections were taken into account while analyzing those specific sections. Frequencies and descriptive statistics were obtained and reviewed. Some of the numeric data were categorized, e.g. age was categorized into 4 categories according to the WHO guidelines [21]. Bi-variate analysis was carried out by cross-tabulating the outcome variables against the socio-demographic variables. Binary logistic regression was conducted to examine the significant correlates of socio-demographic variables with tobacco consumption.

## Results

The socio-demographic characteristics of all the respondents categorized by gender, are shown in Table 1. The mean (± SD) age of the participants was 37.9 (±11.2) years and majority (44.6%) of them were within the 25–34-year age group. Of participants, just above half (50.3%) were males and about half of the respondents (51.5%) had no formal education. Most of the respondents (about 70%) were non-manual workers and majority of them were married (91.7%).

**Table 1:** Socio-demographic characteristics of the adults by gender

Variables	Total n (%) *n* = 507	Male n (%) *n* = 255	Female n (%) *n* = 252
Age Categories (in years)			
Mean ± SD	37.9 ± 11.2	39.5 ± 11.7	36.3 ± 10.4
25–34	226 (44.6%)	107 (42.0%)	119 (47.2%)
35–44	138 (27.2%)	64 (25.1%)	74 (29.4%)
45–54	78 (15.4%)	41 (16.1%)	37 (14.7%)
55–64	65 (12.8%)	43 (16.9%)	22 (8.7%)
Highest Level of Education			
No Formal Education	261 (51.5%)	109 (42.8%)	152 (60.3%)
Below Primary Level	86 (17.0%)	59 (23.1%)	27 (10.7%)
Primary Education completed	62 (12.2%)	31 (12.2%)	31 (12.3%)
Secondary Education and above	98 (19.3%)	56 (21.9%)	42 (16.7%)
Occupation			
Non-manual	354 (69.8%)	117 (45.9%)	237 (94.0%)
Manual	153 (30.2%)	138 (54.1%)	15 (6.0%)
Marital Status			
Never Married	26 (5.1%)	22 (8.6%)	4 (1.6%)
Currently Married	465 (91.7%)	232 (91.0%)	233 (92.4%)
Widowed/Divorced/Separated	16 (3.2%)	1 (0.4%)	15 (6.0%)
Total number of household members			
≤4 members	233 (46.3%)	117 (46.1%)	116 (46.6%)
5 or more members	270 (53.7%)	137 (53.9%)	133 (53.4%)
Monthly Income (in BDT) *			
Interquartile Range (IQR)	IQR = 7500	IQR = 8000	IQR = 8000
≤7500 (≤95 USD)	153 (30.2%)	62 (24.3%)	91 (36.1%)
7501 to 10000 (95 to 127 USD)	117 (23.1%)	70 (27.5%)	47 (18.7%)
10001 to 15000 (127 to 191 USD)	113 (22.3%)	58 (22.7%)	55 (21.8%)
Above 15000 (above 191 USD)	120 (23.7%)	64 (25.1%)	56 (22.2%)

*4 non-responses

The overall tobacco consumption (which includes both smoked and smokeless tobacco) among the adult slum population was 64%. Consumption patterns under each of the following categories, smoking, smokeless tobacco consumption and dual use of tobacco were 35% [95% CI: 31.6–39.8], 40.6% [95% CI: 36.5–45.2] and 12% [95% CI: 9.3–15.0] respectively. The prevalence of tobacco smoking was much higher among the males than the females (Table 2). About 70.6% [95% CI: 65.1–76.08] of the male respondents smoked tobacco as opposed to only 0.4% [95% CI: 0–1.19] of the females. However, consumption of smokeless tobacco (SLT) was more prevalent in females (48.8% vs. 32.6%). Also, among the males 23.5% indulged in dual use of tobacco, i.e. use of both smoked and smokeless tobacco.

**Table 2:** Prevalence of type of tobacco use among the adults by selected demographic characteristics

Variables	Tobacco Smoking % (95% CI)	Smokeless Tobacco Consumption % (95% CI)	Dual Use of Tobacco % (95% CI)
Sex			
Male	70.6 (65.10–76.08)	32.6 (27.06–38.43)	23.5 (18.82–29.01)
Female	0.4 (0.00–1.19)	48.8 (42.47–54.76)	0.4 (0.00–1.19)
Age Categories (in years)			
25–34	34.5 (28.76–40.27)	31.4 (25.22–37.17)	12.0 (7.96–15.93)
35–44	34.1 (26.09–42.03)	47.8 (39.13–56.52)	10.1 (5.07–15.22)
45–54	30.8 (20.51–41.03)	51.3 (39.74–61.54)	12.8 (5.13–20.51)
55–64	49.2 (36.92–61.54)	44.6 (32.31–55.38)	15.4 (7.69–24.62)
Highest Level of Education			
No Formal Education	30.7 (25.30–36.40)	49.0 (42.92–55.16)	10.7 (6.91–14.56)
Below Primary Level	50.0 (39.53–60.47)	44.2 (33.72–54.65)	19.8 (11.63–27.91)
Primary Education completed	35.5 (24.19–48.39)	30.7 (19.35–41.94)	11.3 (3.23–19.35)
Secondary Education and above	36.7 (27.55–46.94)	21.4 (13.27–30.61)	9.2 (4.08–15.31)
Occupation			
Non-manual	22.3 (17.90–27.10)	40.7 (35.80–45.90)	6.5 (4.20–9.30)
Manual	66.7 (58.80–74.30)	40.5 (32.50–48.60)	24.8 (17.80–31.90)
Monthly Income*			
Less than/equal to 7500 (≤95 USD)	27.5 (20.26–33.99)	47.1 (39.22–55.54)	9.8 (5.23–15.03)
7501 to 10000 (95 to 127 USD)	44.4 (35.90–53.85)	42.7 (34.19–52.14)	14.5 (8.55–21.37)
10001 to 15000 (127 to 191 USD)	38.1 (29.20–46.90)	34.5 (26.55–44.25)	11.5 (6.19–17.70)
Above 15000 (above 191 USD)	36.7 (28.35–45.83)	35.8 (27.50–44.17)	13.3 (7.50–19.17)

Among those who used smoked tobacco products, majority (89%) smoked manufactured cigarettes. The mean number of sticks smoked was 13.2 ± 8.3 per day. About 8.3% smoked locally made products such as *bidis* and hookahs. A small proportion (1.1%) smoked a combination of two or more products. Majority (86.9%) of the SLT users consumed *jarda* (a form of chewing tobacco, usually taken with betel quid), while 9.2% consumed *sada pata* (dried plain tobacco leaves) and another 6.8% used *gul* (powdered tobacco, often used as tooth powder for cleaning teeth). Overall 6.8% consumed a combination of two products and 0.97% used all three types.

**Fig. 1:**
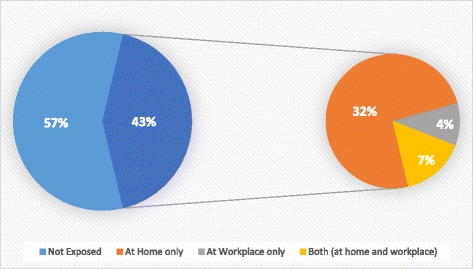
Exposure to second-hand smoking among non-smokers

The exposure status of the non-smokers to second-hand smoking (SHS) at their homes and workplaces have been depicted in Fig. 1. About 43% reported exposure to tobacco smoke, while the rest mentioned that they were not exposed to any form of SHS due to tobacco consumption. Of those exposed, majority (32%) were exposed to SHS at their homes only, while only 4% were exposed to SHS only at their place of work. About 7% experienced SHS exposure both at their homes and workplaces.

The associations between the various types of tobacco consumption and the socio-demographic characteristics were determined by binary logistic regression, the results of which are presented in Table 3, in terms of unadjusted and adjusted odds ratio. The elderly respondents, aged between 55–64 years were twice more likely to smoke (OR: 2.34, 95% CI: 1.21–4.49) than those who were 25–34 years old. The results further revealed that the manual workers were more than eight times (OR: 8.80, 95% CI: 5.48–14.14) more likely to smoke than non-manual workers. For smokeless tobacco, those belonging to the 45–54 years age group (OR: 2.15, 95% CI: 1.24–3.74) were twice more likely to consume SLT than the younger age group of 25–34 years. Also, those who had no schooling history (OR: 2.95, 95% CI: 1.66–5.25) were almost three times likely to use smokeless tobacco than who had higher education (secondary or above). Those who were employed as manual workers were five times more likely to indulge in dual use of tobacco (OR: 5.17, 95% CI: 2.82–9.48) than non-manual workers.

**Table 3:** Logistic regression for the type of tobacco use among the adults residing in urban slums

Socio-demographic variables	Smoking		Smokeless Tobacco		Dual Use	
	Unadjusted	Adjusted#	Unadjusted	Adjusted#	Unadjusted	Adjusted#
	OR (95% CI)	OR (95% CI)	OR (95% CI)	OR (95% CI)	OR (95% CI)	OR (95% CI)
Age Group						
25–34	Ref	Ref	Ref	Ref	Ref	Ref
35–44	0.98 (0.63–1.53)	0.83 (0.49–1.42)	2.00 (1.29–3.10)**	1.68 (1.06–2.67)**	0.83 (0.42–1.65)	0.65 (0.31–1.34)
45–54	0.84 (0.48–1.47)	0.81 (0.42–1.53)	2.30 (1.36–3.89)**	2.15 (1.24–3.74)**	1.08 (0.50–2.36)	0.99 (0.43–2.27)
55–64	1.84 (1.05–3.22)**	2.34 (1.21–4.49)**	1.76 (1.00–3.09)	1.53 (0.85–2.76)	1.34 (0.61–2.94)	1.26 (0.54–2.92)
Education						
No schooling	0.76 (0.47–1.24)	0.49 (0.27–0.89)	3.53 (2.06–6.06)**	2.95 (1.66–5.25)**	1.19 (0.54–2.62)	0.98 (0.41–2.34)
Less than Primary	1.72 (0.96–3.10)*	0.98 (0.49–1.94)	2.90 (1.53–5.52)**	2.84 (1.45–5.57)**	2.44 (1.02–5.80)**	1.66 (0.65–4.25)
Primary completed	0.95 (0.49–1.84)	0.77 (0.36–1.63)	1.62 (0.79–3.34)	1.46 (0.69–3.09)	1.26 (0.44–3.57)	1.11 (0.37–3.32)
Secondary and above	Ref	Ref	Ref	Ref	Ref	Ref
Occupation						
Manual	6.96 (4.58–10.59)**	8.80 (5.48–14.14)**	0.99 (0.68–1.46)	0.79 (0.52–1.20)	4.76 (2.72–8.32)**	5.17 (2.82–9.48)**
Non-manual	Ref	Ref	Ref	Ref	Ref	Ref
Income						
Less than 7500 (≤95 USD)	0.65 (0.39–1.09)	0.48 (0.26–0.88)	1.59 (0.98–2.60)*	1.32 (0.78–2.24)	0.71 (0.33–1.49)	0.53 (0.23–1.20)
7501–10000 (95 to 127 USD)	1.38 (0.82–2.33)	0.92 (0.51–1.68)	1.34 (0.79–2.25)	1.28 (0.73–2.24)	1.10 (0.53–2.31)	0.71 (0.32–1.60)
10001–15000 (127 to 191 USD)	1.06 (0.62–1.80)	0.73 (0.39–1.34)	0.94 (0.55–1.62)	0.84 (0.47–1.48)	0.85 (0.39–1.85)	0.54 (0.23–1.27)
Above 15000 (above 191 USD)	Ref	Ref	Ref	Ref	Ref	Ref

#: variables adjusted in the models: age, education, occupation, income; **: 5% significant, *: 10% significant

## Discussion

The study describes the current status of tobacco use among the adults residing in three urban slums of Dhaka along with associated socio-demographic correlates. Results show that the current tobacco use (both smoked and smokeless forms) among the slum population was higher than the comparable overall tobacco use figures in the national surveys, namely the Global Adult Tobacco Survey (GATS) [6] and the Bangladesh NCD Risk Factor Survey [7]. The overall tobacco consumption (64%) was found to be higher compared to the overall tobacco use in most of the states in neighbouring India [22].

Use of smoked tobacco was more prevalent among the males, which is universally acknowledged [1]. Moreover, compared to the national survey statistics, the findings from the current study implicate that the prevalence of tobacco smoking may be higher in the male slum population. This is complemented by the results of the International Tobacco Control (ITC) Bangladesh Survey 2010, which reports a higher percentage of smokers amongst male slum dwellers as compared to their counter-parts in the rest of the urban population [15]. A previous study (using the results from Urban Health Survey 2006) compared the prevalence of smoking among the males living in slums versus those living in urban areas of Bangladesh and also found a higher prevalence (59.8%) among the slum dwellers as compared to their non-slum counterparts (46.4%) [23]. The present study reports a smoking prevalence of 70.6% among the adult males and thus indicates an increase in male smokers over the years. On the contrary, only 0.4% of the female respondents were found to smoke tobacco which is lower than the NCD risk factor survey report of 1.3% female smokers [7]. Majority of the smokers (97.2%) smoked on a daily basis. While this is consistent with national surveys [6, 7], it is higher than the worldwide estimate of 80% [1].

Majority of the smokers (89%) smoked manufactured cigarettes, which is similar to the figures reported among slum dwellers in the ITC survey [15]. However, this finding is quite different from the findings from several years ago, which reports that 53.3% consumed manufactured cigarettes [23]. This shows a gradual transition towards manufactured cigarettes over the years, most possibly due to greater availability and ease of access to manufactured cigarettes.

In case of smokeless tobacco (SLT), the consumption statistics were higher for both male and female slum dwellers as compared to the national survey reports [6, 7]. The national figures are already high compared to the statistics reported by other SEAR countries like India, Myanmar, Nepal, Sri Lanka and Thailand [24] and even higher rates of SLT consumption among the slum population, particularly the females, are quite an alarming finding. Palipudi et al. (2014) suggested that the reason for this type of trend in SLT consumption among women is possibly because Bangladeshi society is largely conservative and regards smoking as undesirable behavior, especially among females [25]. In contrast chewing SLT products is not viewed as objectionable and thereby the number of females who use SLT are higher [25].

Furthermore, dual use of tobacco (both smoked and SLT) was also higher among the slum dwelling males placing them at a much higher risk of developing NCDs. A study conducted among Bangladeshi men reveals the proportion of dual use of tobacco to be 14.2% [26], which is distinctively lower than the finding from this study. In addition, the manual workers were more likely to indulge in dual tobacco use than non-manual workers. This might be gender moderated and not due to the occupation, as most of the females indulged in non-manual works and majority of the males as manual workers. The gender variable was left out in the models because there was only one female smoker and thus comparisons with the males did not result in any meaningful finding.

Besides direct consumption of tobacco products, second-hand exposure to tobacco smoke can also predispose a person to a relatively higher risk of tobacco-related illnesses, such as ischemic heart disease, stroke and chronic obstructive pulmonary disease, even though they may be non-smokers. Findings from a study show that SHS exposure increases a person’s chances of acquiring any one of these diseases by 21% [27]. Our study reveals that exposure to second-hand smoking (SHS) at home was reported by 39% of the non-smoker respondents. This figure is more or less similar to the values reported in the national surveys (36.3% in the NCD risk factor survey and 43% in the ITC survey) [7, 15].

Age and occupation were found to be significant correlates of tobacco consumption. Age has been identified as a significant correlate of smoking status in many studies [22, 28]. In this study, manual workers were found to be more likely to smoke. This finding is resonated in another study conducted in Bangladesh which reports that people having stressful and laborious jobs were 2.65 times more likely to smoke than those employed in service or business [28]. In the case of smokeless tobacco, education was found to be significantly correlated. Results showed that consumption of SLT went down with higher educational status, which is a reciprocal finding in other developing countries [4].

### Limitations

One of the major limitations of the study was that, there was a possibility of under-reporting of the status of tobacco consumption, since the data collected was self-reported. Information on sensitive topics may have resulted in concealing of correct information, particularly in case of females. Another limitation remains in the fact that the population may not be representative of all the slum population in Bangladesh.

## Conclusion

The increasing problem of tobacco use among the urban slum population has become a major public health threat for Bangladesh. People from low socio-economic condition are more likely to use tobacco and are vulnerable to developing NCDs. Therefore, there is a critical need for developing effective intervention approaches to address the NCD risk factors, thereby preventing the development of NCDs and their consequences.
